# Factors predicting general health concerns and atypical behaviours in children with prenatal alcohol exposure and other adverse exposures

**DOI:** 10.3389/fped.2023.1146149

**Published:** 2023-05-24

**Authors:** Sarah J. MacEachern, Preeti Kar, Daphne Nakhid, Elena Mitevska, Christina Tortorelli, Nils D. Forkert, Catherine Lebel, Carly A. McMorris, W. Ben Gibbard

**Affiliations:** ^1^ Department of Pediatrics, Cumming School of Medicine, University of Calgary, Calgary, AB, Canada; ^2^ Alberta Children’s Hospital Research Institute, University of Calgary, Calgary, AB, Canada; ^3^ Hotchkiss Brain Institute, University of Calgary, Calgary, AB, Canada; ^4^ Cumming School of Medicine, University of Calgary, Calgary, AB, Canada; ^5^ Mount Royal University, Calgary, AB, Canada; ^6^ Department of Radiology, Cumming School of Medicine, University of Calgary, Calgary, AB, Canada; ^7^ Werklund School of Education, University of Calgary, Calgary, AB, Canada

**Keywords:** pediatrics, development, prenatal alcohol (Ethanol) exposure, adverse exposures, health, behaviour

## Abstract

**Background:**

Prenatal alcohol exposure (PAE) can have significant negative consequences on the health outcomes of children. Children with PAE often experience other prenatal and postnatal adverse exposures. Increased rates of general health concerns and atypical behaviours are seen in both children with PAE as well as with other patterns of adverse exposures, although these have not been systematically described. The association between multiple adverse exposures and adverse health concerns and atypical behaviours in children with PAE is unknown.

**Methods:**

Demographic information, medical history, adverse exposures, health concerns, and atypical behaviours were collected from children with confirmed PAE (*n* = 22; 14 males, age range = 7.9–15.9 years) and their caregivers. Support vector machine learning classification models were used to predict the presence of health concerns and atypical behaviours based on adverse exposures. Associations between the sums of adverse exposures, health concerns, and atypical behaviours were examined using correlation analysis.

**Results:**

All children experienced health concerns, the most common being sensitivity to sensory inputs (64%; 14/22). Similarly, all children engaged in atypical behaviours, with atypical sensory behaviour (50%; 11/22) being the most common. Prenatal alcohol exposure was most important factor for predicting some health concerns and atypical behaviours, and alone and in combination with other factors. Simple associations between adverse exposures could not be identified for many health concerns and atypical behaviours.

**Conclusion:**

Children with PAE and other adverse exposures experience high rates of health concerns and atypical behaviours. This study demonstrates the complex effects of multiple adverse exposures on health and behaviour in children.

## 1. Introduction

Prenatal alcohol exposure (PAE) is one of the most common in-utero adverse substance exposures worldwide (1) and it is well documented that PAE has a significant negative impact on the developing brain (2, 3). When PAE occurs at a sufficient frequency and/or volume, it can result in fetal alcohol spectrum disorder (FASD), which is a heterogenous neurodevelopmental disability affecting approximately 2%–5% of the North American population (4). Neurodevelopmental domains that can be impacted include neuroanatomy (i.e., microcephaly), cognition, language, memory, executive function, adaptive behaviour, and social and motor skills (5). Children with PAE and FASD are also at higher risk of physical, neurodevelopmental, and mental health disorders, including conductive or sensorineural hearing loss, intellectual disability, attention deficit hyperactivity disorder (ADHD), anxiety disorders, and psychosis (3, 6).

Children with PAE and FASD can also have health concerns that are not formal medical diagnoses, such as difficulties falling or staying asleep or differences in bowel or bladder function (3, 7–10). Differences in sensory perception have also been reported in children with PAE and FASD, such as hyper- or hypo-sensitivity to noises, sounds, or smells (11, 12). Sensory differences can also lead to health concerns, such as decreased sleep quantity and overnight awakenings (10). These health concerns and sensory differences have the potential to significantly impact these children and their families’ lives. However, these health concerns may be missed or misdiagnosed within the medical system and are often not taken into consideration in the majority of research studies involving children with PAE; consequently, systematic studies describing the health concerns of children with PAE are limited.

Children with PAE and FASD can also display a range of uncommon or unusual behaviours that are often socially inappropriate, such as eating non-food items (pica), hoarding food despite adequate access, and toileting behaviours such as defecating outside of the washroom or fecal smearing (9, 13–15). For the purpose of this study, we have used the term “*atypical behaviours*” to describe these types of uncommon, unusual, or socially inappropriate behaviours. Other reported atypical behaviours include sensory elements such as rubbing hair, biting themselves, sucking fingers, chewing clothing, fondling own genitals, and masturbation, which we refer to as “*atypical sensory behaviour*.” Most studies investigating atypical behaviours in children with PAE focus on one specific behaviour, such as abnormal eating (13, 14) or use a qualitative methodological approach (15). Atypical behaviours have not been systematically studied in this patient population.

PAE typically does not occur in isolation; children with PAE often experience multiple adverse exposures *in utero* in addition to alcohol, such as exposure to other harmful substances (tobacco, cannabis, cocaine, methamphetamines, opioids, *etc.*) or maternal toxic stress (16, 17). Children with PAE are at a higher risk of adverse childhood experiences than their peers (18, 19) such as witnessing or experiencing violence, abuse, or neglect, and these have dose-dependent and cumulative negative effects on physical and mental health outcomes over the lifespan (20–22). It is difficult to separate the relative contributions of each influence on the child's overall development (16, 23). However, the presence of multiple adverse exposures in early childhood is associated with worse developmental outcomes (16). For example, having both PAE and traumatic childhood experiences puts children at higher risk for difficulties with cognition, language, attention, memory, and behaviour than children with one of those exposures alone (23–25). Quantifying exposures is therefore important, and we utilized a risk characterization framework developed to assist clinicians and researchers to categorize and rank prenatal and postnatal adverse exposures with accuracy and consistency (17). This framework has not previously been utilized to explore health concerns and atypical behaviours in children with PAE and other adverse exposures, and the relationship between these outcomes is not well understood.

Supervised machine learning aims to identify complex patterns within high-dimensional data which can be used to classify or make predictions in new patients (26). Machine learning has been utilized in numerous clinical and research applications investigating interactions in human health and disease (27). Given the complexities of prenatal and postnatal exposures and adverse experiences, machine learning is well suited to identify complex relationships in data; however, machine learning has not previously been applied to study children with PAE and other adverse exposures.

Addressing these knowledge gaps, the present study described health concerns and the presence of atypical behaviours in children with PAE and other adverse prenatal and postnatal exposures. Children with PAE and multiple adverse exposures were anticipated to have a high frequency of health concerns and atypical behaviours. Second, we utilized machine learning models and identified patient-specific adverse exposures that can be used to predict the presence of health concerns and atypical behaviours. To the best of our knowledge, this study is the first to look comprehensively at common elements of the health history in children with PAE and other adverse exposures, including formal diagnoses, health concerns, and atypical behaviours. This study is also the first to use machine learning to predict specific health concerns and atypical behaviours based on the adverse exposures experienced in this population with the goal of impacting clinical assessments and understanding of individuals with PAE.

## 2. Materials and methods

### 2.1. Ethical approval

Ethics approval was obtained from the Conjoint Health Research Ethics Board (CHREB) of the University of Calgary, Calgary, AB (REB 17-0663). Caregivers and youth provided written informed consent and assent, respectively. All study protocols and procedures were conducted in compliance with the Declaration of Helsinki. Data was stored on secured servers. All documents were password-protected.

### 2.2. Participants

A total of 22 children and adolescents with PAE between 7 and 16 years of age (10.7 ± 2.4 years; 7.92–15.98 years; 14 males/8 females) were recruited from a tertiary developmental clinic, Children's Services, online advertisements, caregiver support groups, and word of mouth in Alberta, Canada between Spring 2017 and Summer 2019. Exclusion criteria for participants were birth prior to 34 weeks gestation, a history of head trauma, youth for whom English was not a primary language, and a diagnosis of a medical or genetic disorder associated with serious motor or cognitive disability (e.g., autism, epilepsy, cerebral palsy). Participants with ADHD, learning disabilities, and/or mental health diagnoses were included, as these frequently co-occur with PAE. All children and adolescents were invited to the Alberta Children's Hospital for comprehensive assessments [results published elsewhere (28)] and caregiver surveys.

### 2.3. Measures

Information about the child's prenatal and postnatal exposures were collected from child welfare files and/or from semi-structured interviews with current caregivers, caseworkers, and/or birth families. Specific measures, which have been described in detail elsewhere (17) included PAE, other prenatal substance exposure (i.e., nicotine, cannabis, cocaine, methamphetamines, opioids), prenatal maternal mental health diagnoses, prenatal maternal neurodevelopmental disabilities (NDDs), prenatal toxic stress (i.e., lack of prenatal care, housing, food, or income to meet needs), prenatal fetal trauma, prenatal maternal adverse childhood experiences (ACEs), experiencing postnatal threat (harm or threat of harm including physical, emotional, sexual abuse, or witnessing violence, substance abuse, or criminal activity in the home) from 0 to 24 months or from 25 months onwards, and experiencing postnatal deprivation (the basic needs of the child not being met or a risk of needs not being met) from 0 to 24 months or from 25 months onwards. Each measure was ranked within the risk characterization framework as previously described (17). For example, PAE was ranked on a scale from 1 to 4, where 1 is confirmed absence of exposure, 2 is unknown exposure, 3 is exposure to prenatal alcohol not meeting criteria for a score of 4 *or* confirmed exposure of unknown amount, and 4 is high exposure of 7 or more drinks per week or 2 or more binge episodes of 4 or more drinks at some point in the pregnancy. Caregivers also completed a demographic survey reporting their relationship to the child, their child's age and sex, and household income (collected categorically: <$25,000; $25,000–49,999; $50,000–74,999; $75,000–99,999; $100,000–124,999; $125,000–149,999; $150,000–174,999; >$175,000).

The health concerns and atypical behaviours survey (Supplementary Material) was created by adapting a screening questionnaire designed to identify children with “special health care needs” (29) for use in children with PAE. This was supplemented with input from individuals with clinical experience working with pediatric patients who have complex exposures and atypical behaviours, and a literature review. The survey was validated through a face and content validity review by clinicians and researchers working with children with PAE and FASD. Medical conditions were reviewed by a developmental pediatrician (SJM) to ensure accuracy and standardized terminology. All responses were anonymized.

### 2.4. Data analysis

Machine learning analysis was conducted using an in-house machine learning pipeline described elsewhere (30, 31). Briefly, the machine learning pipeline consists of a feature ranking method followed by training of a supervised machine learning method. First, all available features (patient-specific adverse exposures) were automatically ranked with respect to their relative importance for each outcome measurements (health concerns and atypical behaviours) using the RreliefF feature ranking algorithm. This algorithm ranks the feature with respect to its importance for the outcome measurement and accounts for inter-variable correlations, so that only highly informative and non-redundant features are ranked highly (32). The ranked features were then used for training of a linear kernel support vector machine (SVM). SVMs were used for this purpose as they are known to perform well, even in case of small sample sizes available for training. The linear kernel together with a standard cost parameter of 1.0 were selected for this purpose to prevent overfitting of the model. The SVM model was trained on iteratively reduced feature sets. The least informative feature as determined by the RreleifF feature ranking was removed from the feature set in each iteration, which was then used for retraining of the SVM. This procedure was conducted until only a single feature was left for training of the SVM. In order to determine the optimal feature subset (i.e., number of features), the area under the receiver operating characteristic curve (ROC-AUC) was used to compare the models. In case two or more models for the same outcome measurement had the same ROC-AUC value, the model with the lower number of features was selected as the best model following Occam's Razor. This procedure was repeated separately for each outcome measure. Each model was trained and evaluated using a leave-one-out cross validation to maximize the available training set in each iteration. The feature ranking was only performed based on the training set in each iteration, to prevent double dipping. For each outcome, the results of the best performing machine learning model as evaluated by the ROC-AUC are reported, including the ROC-AUC value, the accuracy, as well as the optimal subset of features used by each model.

The machine learning pipeline was also used to predict summary health and behaviour outcomes based on patient-specific adverse exposures. The number of health concerns and the number of atypical behaviours were summed for each patient. The summary scores for the outcomes, which were the number of health concerns and the number of atypical behaviours, were then binarized into two groups (“low” vs. “high”) with the aim to achieve optimally balanced group sizes (as similar as possible in number). This approach is beneficial for machine learning models, which are known to perform best when trained with balanced group sizes. The health concern score was optimized by defining “low” as patients with four or less health concerns and all other children as “high”. For the atypical behavior summary score, “low” was defined as three or less atypical behaviours and all other children as “high”.

A machine learning model with a ROC-AUC value of 0.5–0.6 was considered a fail, a value of 0.6–0.7 was considered poor, a value of 0.7–0.8 was considered fair, a value of 0.8–0.9 was considered good, and a model with a value of 0.9–1.0 was considered excellent. Only classifiers with a ROC-AUC value of 0.7 or better and with an accuracy better than chance level were considered successful and clinically relevant. Models with a ROC-AUC of 0.6–0.7 and with an accuracy higher than chance level were considered potentially clinically relevant. All other models were considered clinically irrelevant and are not discussed in detail in the Results.

The association between the number of PAE and adverse exposures, the number of health concerns, and the number of behavioral problems were investigated using Pearson correlation coefficient. A correlation with a *p*-value <0.05 was considered significant.

## 3. Results

### 3.1. Demographic and health data

For demographic data, the neurodevelopmental, medical, and mental health diagnoses, and the body mass index (BMI) of participants, please refer to Table 1. The average age of placement into a permanent stable home was 2.2 ± 3.4 years (0–12 years). The median household income was $100,000–$124,999 CAD (∼$78,000–$97,500 USD). BMI was calculated for each child based on the CDC growth charts (33).

**Table 1:** Demographic data of study participants, child medical and mental health diagnoses, and BMI.

Demographic information	*n* (*x*/22)	Percentage
**Self-Identified Ethnicity**		
White	10	46%
Indigenous	6	27%
Multiracial	4	18%
Not disclosed	2	9%
**Place of residence**		
With adoptive parents	18	82%
With biologic grandparents	3	14%
With foster caregivers	1	4%
Biologic parents	0	0%
Type of diagnosis	*n* (*x*/22) & Percentage	Specific conditions or Patient profile
FASD or at risk of FASD	9 (41%)	– FASD—8 – At Risk of FASD—1
Neurodevelopmental disorders	17 (77%)	– ADHD—14 – LD—6 – DCD—1
Mental health conditions	6 (27%)	– Anxiety—4 – PTSD—1 – Conduct disorder—1
Physical health conditions	14 (64%)	– Skin condition such as psoriasis or atopic dermatitis (eczema)—6 – Asthma—4 – Allergies—2 – Migraines—2 – Tourette's—1 – Scoliosis—1 – Nocturnal enuresis—1 – Febrile seizures—1 – Arthritis/Arthralgias—1 – Congenital malformation of the foot—1 – Cleft lip—1
Child weight status and body mass index (BMI)	*n* (*x*/22)	Percentage
Underweight (BMI <5th percentile)	1	5%
Healthy weight (BMI 5–85th percentile)	16	73%
Overweight (BMI >85th percentile)	4	18%
No data	1	5%

ADHD, attention deficit hyperactivity disorder; LD, learning disability; DCD, developmental coordination disorder; PTSD, post-traumatic stress disorder, BMI, body mass index.

### 3.2. Complex exposures of children

Most participants had confirmed PAE (95%). 11 participants (50%) had confirmed exposure meeting the Canadian diagnostic guidelines for FASD [(5); ≥7 drinks/week or ≥2 binge episodes of at least 4 drinks at some point in pregnancy]; see Table 2 for details of PAE and other adverse exposures.

**Table 2:** Documented exposures of study participants.

Exposure	*n* (*x*/22)	Percentage
**Prenatal alcohol exposure**		
High PAE (≥7 drinks per week or ≥4 drinks on one occasion)	11	50%
Confirmed PAE (below threshold for high PAE)	10	45%
Unknown exposure	1	5%
No exposure	0	0%
**Prenatal exposure to other substances**		
High frequency of use (≥5 in pregnancy)	9	40%
Exposure to nicotine or cannabis at any point; low frequency of use of other substances (cocaine, opioids, *etc*) or confirmed use of unknown amount	11	50%
Unknown exposure	2	10%
No exposure	0	0%
**Maternal psychosocial stressors**		
Any maternal psychosocial stressor	22	100%
• Prenatal maternal mental health diagnoses	9	41%
• Prenatal maternal NDDs	4	18%
• Prenatal toxic stress—deprivation	14	64%
• Prenatal toxic stress—threat	13	59%
• Prenatal fetal trauma	1	5%
• Prenatal maternal ACEs	11	50%
**Postnatal adversity**		
Any postnatal adversity	20	91%
• Postnatal exposure to threat from 0 to 24 month	9	41%
• Postnatal exposure to threat from 25 month+	8	36%
• Postnatal exposure to deprivation from 0 to 24 month	16	73%
• Postnatal exposure to deprivation from 25 month+	6	27%

NDDs, neurodevelopmental disabilities; PAE, prenatal alcohol exposure; m, months; +, onwards.

### 3.3. Caregiver-reported health concerns and atypical behaviours of children with PAE and Complex exposures

The majority of caregivers rated their child's overall health positively, with 95% rating their child's health as “good”, “very good”, or “excellent”. One caregiver “didn’t know” and no caregivers rated their child's health negatively. All children were reported to have a health concern (Table 3) and were reported to engage in at least one atypical behaviour (Table 4).

**Table 3:** Health concerns of children as reported by parents.

Health concern	*n* (*x*/22)	Percentage and specific conditions or Patient profile
Presence of any health concern	22	100%
**Hearing and Vision**		
Hearing difficulties	4	18%
History of ear infections	9	41%
Vision difficulties	13	59%
		– Nearsighted—9/22 – Farsighted—2/22 – CNS visual processing concerns—2/22
**Bowel habits**		
Constipation—current and past history	8	36%
Diarrhea or loose stools unrelated to infection—current or past history	1	5%
**Sleep difficulties**		
Difficulties falling asleep two or more nights per week	12	55%
		Reasons for difficulties falling asleep
		– Cannot stop mind from going—4 – Worry—4 – Medication side effect—3 – Hunger—1
Overnight waking more than two nights per week	10	45%
		Reasons for overnight waking
		– Going to the bathroom—6 – Worry/anxiety—4 – Restless—3 – Getting food—1
**Sensory symptoms**		
Sensitivity to sensory inputs	14	64%
		Specific sensory input
		– Sound—4 – Touch—4 – Sound, Touch—4 – Sound & Lighting—1 – Sound, Lighting, and Touch—1
**Pain experiences**		
Frequently experience pain	8	36%
Having a high pain tolerance	15 Yes	68%
Usual pain intensity	Mild—11	
	Moderate—3	
	Severe—3	
	Don’t know—2	
	No response—3	
Activity limitations secondary to pain	None—17	
	A Few—4	
	Some—1	
	Most—0	
	Don’t know—0	
**Serious injury**		
History of serious injury^a^	8 Yes	36%

CNS, central nervous system.

^a^ Of note, the study exclusion criteria included a history of head trauma.

**Table 4:** Child atypical behaviours as reported by parents.

Health concern	*n* (*x*/22)	Percentage
Presence of any atypical behaviours	22	100%
**Sensory behaviours**		
Engaging in atypical sensory behaviour	11	50%
Skin picking and excoriation	6	27%
Trichotillomania	1	5%
**Food & dietary behaviours**		
Eating non-nutritive substances at present or in the past	6	27%
Hoarding objects at present or in the past	8	36%
Hoarding food	7	32%
Taking food without permission outside of mealtimes	8	36%
Needing to lock the pantry or fridge to keep the child from accessing food	4	18%
Continuing to eat despite being full	5	23%
Eating to the point of throwing up	4	18%
Lack of interest in food	5	23%
Picky eater	8	36%
**Toileting behaviours**		
Intentionally urinating in places other than the bathroom	3	14%
Having bowel movements in places other than the bathroom	2	9%

### 3.4. Prediction of health concerns and atypical behaviours using exposure data

The overall best results were achieved by the machine learning model predicting hearing problems based on adverse exposures. This machine learning model achieved an accuracy of 91% using a combination of variables including experiencing early threat from 0 to 25 months and deprivation from 25 months onwards, prenatal fetal trauma, prenatal maternal mental health diagnoses, prenatal maternal ACEs, prenatal maternal NDDs, and prenatal toxic stress. The corresponding ROC-AUC of this model was 0.75.

Having a history of ear infections could be predicted with an accuracy of 64% by the optimal machine learning model based on prenatal toxic stress and PAE. However, the classifier had a poor ROC-AUC (0.624).

The optimal SVM model was able to predict children taking food outside of mealtimes with an accuracy of 77% based on PAE alone (ROC-AUC = 0.795) and hoarding food with an accuracy of 73% based on deprivation and threat after 25 months, prenatal maternal NDDs, PAE, prenatal substance exposure, prenatal fetal trauma, prenatal toxic stress, and threat between birth and 24 months, although the ROC-AUC was in the poor range (0.686).

The optimal machine learning model predicting difficulties with overnight waking during sleep used PAE as the only feature. Using this one variable, the classifier achieved an accuracy of 77% and a ROC-AUC value of 0.78. The best classifier for predicting difficulty falling asleep used the presence of prenatal maternal ACEs as the only feature and achieved a classification accuracy of 64% but a poor ROC-AUC value (0.642).

The best performing machine learning model for predicting a high tolerance to pain achieved an accuracy of 73% using a combination of variables, including prenatal maternal mental health, experiencing threat from 0 to 24 months of age, prenatal maternal ACEs, prenatal maternal NDDs, prenatal substance exposure, and experiencing deprivation at any age. However, the ROC-AUC was in the poor range (0.69).

Based on the features available, the machine learning models were not able to successfully predict any other outcomes as indicated by ROC-AUC values lower than 0.6 and accuracies below the chance level.

The best performing SVM was able to predict the binarized health concerns outcome measurements (i.e., health concerns in “high” or “low” range) with an accuracy of 68% and ROC-AUC value of 0.662. This optimal classifier used a combination of variables including PAE, experiencing deprivation from 0 to 24 months, experiencing threat at any age, prenatal fetal trauma, prenatal maternal NDDs, prenatal maternal ACEs, and prenatal toxic stress. The best performing SVM model for predicting children with a high or low summary behavioral score (i.e., the presence or absence of four or more atypical behaviours) achieved an accuracy of 64% and ROC-AUC of 0.624 using a combination of PAE, prenatal maternal mental health diagnoses, prenatal toxic stress, prenatal substance exposure, prenatal fetal trauma, prenatal maternal NDDs, and experiencing threat beyond 25 months.

**Figure 1:**
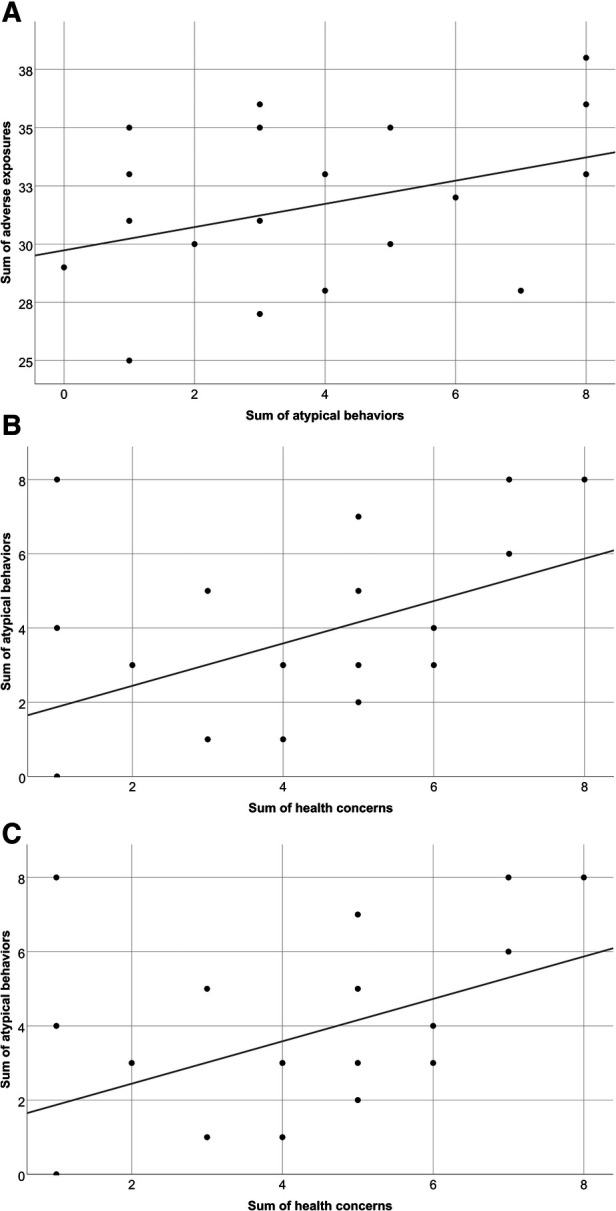
Scatter plots demonstrating comparisons between the summary scores of (**A**) adverse exposures and health concerns; (**B**) adverse exposures and atypical behaviours; and (**C**) health concerns and atypical behaviours for children with PAE and other adverse exposures.

The statistical analysis comparing the summary scores of adverse exposures and the sum of health concerns showed no correlation (*r* = 0.084, *p* = 0.710). Similarly, only a weak but non-significant positive correlation was found comparing the sum of adverse exposures and sum of atypical behaviours (*r* = 0.364, *p* = 0.095). However, there was a modest significant correlation comparing the sum of health concerns and the sum of atypical behaviours (*r* = 0.451, *p* = 0.035) indicating that children with more health concerns were more likely to display more atypical behaviours (Figure 1).

## 4. Discussion

In this study we show that in a group of children with PAE and other adverse exposures, all children experienced health concerns and engaged in atypical behaviours. Health concerns related to sensitivities to sensory inputs and atypical behaviours related to atypical sensory behaviours were most reported. Using machine learning techniques, PAE alone and in combination with other adverse exposures was identified as the most predictive factor for classifying the presence of specific health concerns or atypical behaviours. However, many health concerns or atypical behaviours could not be predicted with high accuracy based on adverse pre- and post-natal exposures alone. This study reinforces the complexity of understanding the impact of multiple adverse exposures on health and behaviour.

Children in this study had a neurodevelopmental and medical profile similar to that observed in prior studies of children with PAE or FASD. Many had at least one diagnosis of a co-occurring neurodevelopmental condition, most commonly ADHD and learning disabilities, consistent with profiles of children with PAE and FASD described elsewhere (3). Anxiety was the most diagnosed mental health condition, which also aligns with the literature (3). Many participants had a diagnosed physical health condition, the most common being a skin condition such as psoriasis or atopic dermatitis (eczema), which again corresponds with existing literature (34). Therefore, the children in this study have a neurodevelopmental and medical profile representative of what is typically observed in children with PAE and FASD in other studies.

In this study, all children had general health concerns that are less easily captured in medical records as they are not part of a formal health or neurodevelopmental diagnosis, such alterations in sleep or difficulties with bowel movements. These health concerns could result in significant challenges for the child and their family. For example, disruptions in sleep—impacting up to 85% of children with FASD (8, 10)—can negatively impact a child's physical health, behaviour, mental health, academic abilities, and cognitive development. It can also have harmful consequences on family functioning including negative effects on the physical and mental health of parents and caregivers (35, 36). Additionally, all children in this study had atypical behaviours, which is in line with other studies of children with FASD which have looked at subsets of atypical behaviour (9, 13–15). Atypical behaviours can have a significant impact on these children and their families, such as limiting participation in school, extracurricular activities, and their community, especially when exhibiting behaviours that are socially inappropriate in nature (e.g., masturbation or defecation outside of the bathroom).

Most children experienced sensitivities to sensory inputs and atypical sensory behaviour, suggesting that sensory differences play a significant role in the lives of children who have PAE and a history of complex exposures. This connection is supported by previous literature, where others have documented differences in sensory and auditory processing and hypo- and hyperresponsiveness to sensory stimuli from every sensory domain (9, 11, 12). The pathophysiology of sensory differences in FASD has been postulated to originate from the impact of in-utero exposure to alcohol on sensory neurons and brain regions involved in the perception of sensation, including touch, pain, smell, taste, vision, and hearing (3, 9). Neuroimaging studies have identified structural brain differences in children with FASD compared to controls in regions involved in sensory perception and processing, including the cortex, corpus callosum, basal ganglia, hippocampus, uncinate fasciculus, and the pyramidal tract of the motor system, as well as reduced functional connectivity (37–39). Children with PAE and other adverse postnatal exposures have distinctive structural brain differences compared to those with PAE alone using brain scans from neurotypical children as a reference, in particular within the cortex and limbic system, suggesting that postnatal exposures moderate the effects of PAE on the developing brain (28). Prenatal alcohol has also been postulated to have significant adverse effect on the hypothalamic-pituitary-adrenal (HPA) axis, which is part of the neuroendocrine system involved in the regulation of stress, appetite, and the autonomic nervous system. PAE can alter the HPA axis, which may alter an individual's response to external stressors (40, 41). For example, in infants and toddlers, PAE may blunt the body's stress response to pain but enhance the response to social stress (40). Therefore, altered perception and processing of sensory stimulation and regulation of external stressors via the HPA axis may drive the sensory differences and atypical behaviour observed in this study.

One of the most notable of the atypical behaviours is the intentional urination and bowel movements outside of the bathroom and fecal smearing. Most commonly, atypical toileting is suggestive of gastrointestinal dysfunction such as constipation and abdominal pain, which may or may not have an organic cause (42). High rates of gastrointestinal concerns have been described in both this cohort and others, with constipation, diarrhea, and other gastrointestinal symptoms occurring with a far higher frequency in those with FASD than those without (9). The cause of this is unclear and is likely multifactorial from a combination of factors including dietary, behavioural, and medical, among others (42). Fecal soiling has historically been considered to be a red flag for child sexual abuse, although more recent evidence has called this correlation into question, finding in a large sample of children that fecal soiling was not predictive of a history of child sexual abuse and instead more likely related to typical gastrointestinal dysfunction in particular constipation (43). No children in our study who experienced sexual abuse (categorized under postnatal exposure to threat) engaged in atypical toileting behaviours.

With data from a framework to categorize and comprehensively rank prenatal and postnatal adverse exposures (17), the machine learning models were able to predict some health concerns and atypical behaviours using information about specific pre- and post-natal adverse exposures. PAE was a strong predictor of many outcomes, for example being identified as the sole factor influencing overnight waking during sleep and taking food outside of mealtimes. These findings are not unexpected based on previous literature, as the link between disordered sleep and PAE is well documented and may reflect circadian and homeostatic variations secondary to the effects of alcohol on the developing brain (8, 10). Similarly, atypical food related behaviours have previously been documented in children with PAE (13, 14). However, some of the other findings are difficult to interpret in the context of previous literature. For example, the results achieved by the machine learning model related to hearing problems did not identify PAE to be predictive, but primarily selected maternal and prenatal factors including prenatal maternal psychosocial stress, prenatal toxic stress, prenatal fetal trauma, *etc.* and postnatal threat and deprivation. This is in contrast to the other machine learning models predicting hearing-related problems in this study including a history of ear infections and sensitivity to sound, which selected PAE as an informative feature. Similarly, other studies have found a strong association between PAE and hearing differences (3). Overall, the majority of health concerns and atypical behaviours could not be predicted by the machine learning models, which may seem surprising given the well-documented and strongly teratogenic effect of prenatal alcohol. This may be explained in part by other confounding variables such as genetic predisposition, nutrition, and health care support that was not collected in this study.

The machine learning models used to predict high levels of health concerns or atypical behaviours showed considerable overlap in the selected adverse exposures (specifically PAE, prenatal fetal trauma, maternal NDDs, prenatal toxic stress, and experiencing threat beyond 25 months). Some factors were only selected by the machine learning model for prediction of high levels of health concerns (threat and deprivation from 0 to 24 months, prenatal maternal ACEs) while others were only selected by the model predicting high levels of atypical behaviours (prenatal maternal mental health, prenatal substance exposure). Only one factor—deprivation beyond 25 months—was not selected by any of the two machine learning models as being predictive for the outcomes. This suggests that, while it was difficult to untangle the specific effects of each adverse exposure, almost all adverse exposures had an impact on the health and behaviour outcomes explored in our study.

In theory, machine learning models might be able to untangle these complex relationships using more advanced explainable artificial intelligence and causal analysis methods. The difficulties faced by our machine learning model may be due in part to the small sample size, the large number of adverse exposures within the sample, and the lack of a control group for comparison (see *Study Limitations* below for further details). However, these results also highlight that exposure to specific adversities does not necessarily determine clinical or functional challenges, even in a sample of children with a large burden of adversity in early life, as supported by the weak correlation between the sum of adverse exposures and health concerns or atypical behaviours. This suggests that the risk of multiple adverse exposures on health and behaviour is not captured by their simple summation but is a more complex relationship between the single exposures, including factors such as timing, dose, duration, and interaction of specific substances, among others.

Another important consideration is that the data collected in this study did not include protective factors, such as personal resilience or the presence of a loving caregiver or stable environment. Often the approach to clinical work and research investigating PAE, FASD, and adverse exposures is deficit and impairment focused, and it is essential to remember the contribution of positive influences in the lives of these children. In support of this approach is that—despite the challenges described above—most caregivers rated their child's health positively. Exploring modifiable protective factors for health and developmental outcomes in children with PAE is an important area of future research.

### 4.1. Study limitations

This study has several limitations. First, the sample size is small and data collection occurred in a single city. Therefore, our findings may not be generalizable for all children with PAE, FASD, and other adverse exposures or to other regions. Second, the study uses a non-standardized non-normed survey, which may limit reproducibility; however the survey was developed based on other published surveys and content was approved by experts in the field. The survey can be found in the Supplementary Material and we welcome free use of the survey by other groups. Third, the exposures were quantified to the best of our ability based on historical data, but frequently did not have highly specific details about timing, doses, frequency of exposures. This data may also be missing other exposure information as information may not have been reported/available within the source materials. This is not unique to our study—a major challenge of clinical research involving children who had multiple adverse exposures it that it is difficult to accurately quantify each exposure based on historical information. Fourth, our study did not investigate the impact of health concerns or atypical behaviours on the child's well-being, functioning, or quality of life at home, school, and other environments. Therefore, we do not have a measure of the impact of these challenges on the child or their family. This represents an important future direction of this work.

### 4.2. Clinical pearls for medical professionals working with children with PAE and other adverse exposures

There are several important clinical take-aways from this study. First, the ubiquitous nature of health concerns and atypical behaviours amongst children in this study illustrates the importance of conducting a detailed health history and behavioural assessment for children with PAE. An exploration of health concerns and atypical behaviours will likely provide further information that may not be captured in formal diagnoses, and may have significant negative impacts on the functioning of the child and their family. We suggest screening for health concerns and atypical behaviours as part of standard care for children with PAE, especially if they have a history of other adverse exposures. Second, atypical behaviours can often be difficult to understand and treat. This study suggests that having more health concerns is associated with having more atypical behaviours, and indeed could underlie challenging behaviour in children with FASD and other adverse exposures. Therefore, treatment of health concerns could be a therapeutic treatment target to help ameliorate atypical behaviours. For example, investigating and treating sleep problems could have a significant positive impact on the child and their family (e.g., mental health, quality of life, well-being), and should not be ignored. Additionally, if typical “starting points” for clinical treatment fail (such as treating co-occurring ADHD), our results suggest that next steps should include exploring sensory differences and targeting those challenges environmentally, behaviourally, and with psychopharmacology as potential therapeutic targets for treating behavioural challenges. Third, children may present to their family doctor, pediatrician, or other health care provider with a primary complaint of challenging behaviour, and the clinician should ensure a thorough assessment for prenatal and postnatal adverse exposures is conducted to rule out PAE or other adverse experiences which may underlie health differences and behavioural challenges. Finally, a clinical approach to children with PAE and complex exposures should also consider positive and protective factors, as these are often overlooked and may help the patient, family, and health care team overcome the stigma that often accompanies these diagnoses.

## 5. Conclusion

Children with PAE and other adverse prenatal and postnatal exposures are at considerable risk of health problems and atypical behaviours, in particular challenges related to sensitivities to sensory inputs and atypical sensory behaviour. Prenatal alcohol exposure alone and in combination with other adverse exposures may contribute the presence of specific health concerns or atypical behaviours and should be explored in the evaluation, care, and treatment of these children. The findings in this study, including the convoluted relationship between exposures, reinforces the complexity of multiple adverse exposures on health and behaviour in children and youth with PAE and FASD. Additionally, it highlights the importance of protective and other positive factors in the lives of children and youth with PAE and FASD, which must be recognized and celebrated.

## Data Availability

The raw data supporting the conclusions of this article will be made available by the authors, without undue reservation.
