# Supplementary material for: Factors predicting general health concerns and atypical behaviours in children with prenatal alcohol exposure and other adverse exposures
Source: Front Pediatr. 2023 May 24;11:1146149. doi: 10.3389/fped.2023.1146149 (PMC10244621; doi:10.3389/fped.2023.1146149)
Supplement: Supplementary file 1 [file Datasheet1.docx]

**Physical Health Questionnaire**

*Please complete the following questions regarding any physical health issues your child may have. Please complete as accurately as possible. Your information will be kept confidential.*

**General Health**

1. What is your child’s height? _________________
2. What is your child’s weight? _________________
3. In general, would you say your child’s health is:
   1. Excellent
   2. Very good
   3. Good
   4. Fair
   5. Poor
   6. Don’t know
4. Describe some of your child’s current health related issues:
   ________________________________________________________________________________________________________________________________________________
5. Does your child currently have any long-term health conditions:
   1. Food or digestive allergies
   2. Asthma
   3. Respiratory allergies such as hay fever
   4. Any other allergies
   5. Bronchitis
   6. Diabetes
   7. Heart condition or disease
   8. Epilepsy
   9. Cerebral palsy
   10. Kidney condition or disease
   11. Migraines
   12. Psoriasis/eczema, or any other skin condition
   13. Any other long-term condition
   14. None
   15. OTHER (please specify): _______________________

1. Does your child CURRENTLY have constipation? YES / NO
2. In the PAST, has your child had constipation? YES / NO
3. Does your child CURRENTLY frequently have loose stools or diarrhea (not related to an acute infection) ? YES / NO
4. In the PAST, has your child had loose stools or diarrhea (not related to an acute infection) ? YES / NO
5. Does your child struggle to fall asleep at night (2 or more times during the week)? YES / NO
   1. If YES, what are some reasons that you think your child may not be able to sleep at night?
      1. Rumination
      2. Worry
      3. Cannot stop their mind from going
      4. Hungry
      5. Other: _________________
      6. Not Applicable

Does your child frequently wake in the night (more than 2 times per week)? YES / NO

a. If YES, what are some reasons that you think that your child may frequently wake through the night?

Nightmares

Night terrors

Goes to bathroom

Worry/Anxiety

Restless

- - 1. Apnea/stop breathing
    2. Other

1. Is your child especially sensitive to sensory inputs?
   1. Touch (i.e., sensitive to tags in clothes or feel of different fabrics)
   2. Lighting
   3. Sounds
2. Does your child have a high pain tolerance? YES / NO
3. Does your child frequently complain of pain or discomfort? YES / NO
4. How would you describe the usual intensity of your child’s pain or discomfort?
   1. Mild
   2. Moderate
   3. Severe
   4. Do not know
5. How many activities does your child’s pain/discomfort prevent them from doing?
   1. None
   2. A few
   3. Some
   4. Most
   5. Do not know
6. In the past, was your child injured seriously enough to require medical attention from a doctor, nurse, or dentist? YES / NO

**Hearing and Vision:**

1. Has your child had their hearing checked by an audiologist? YES / NO
2. Is your child hard of hearing? YES / NO
3. Since birth, has your child had an ear infection? YES / NO
   1. If YES, please specify how many times: _________________________
4. Is your child overly sensitive to sound? YES / NO
5. Has your child had their vision checked by an optometrist or ophthalmologist? YES / NO
6. Does your child have any vision issues?
   1. Nearsighted
   2. Farsighted
   3. Lazy eye/Strabismus
   4. Amblyopia
   5. Other: __________
   6. N/A

**Less frequent/Atypical behaviours**

*Sometimes children/youth will engage in behaviours that can be considered atypical or odd. Some of these questions may not apply to you or your child.*

1. Does your child CURRENTLY eat substances that have no nutritional value (such as dirt, paper, or paint)? YES / NO
   1. If YES, how often? ________________
2. What substances? _________________In the PAST, has your child ever ate substances that have no nutritional value (such as dirt, paper, or paint)? YES / NO
   1. If YES, how often did this occur? ____________________
   2. What substances? ______________________
3. Does your child CURRENTLY hoard, stock, or store objects? (e.g., wrappers, money, paper) YES / NO
4. In the PAST, did your hoard, stock, or store objects? (e.g., wrappers, money, paper) YES / NO
5. Does your child CURRENTLY engage in any self-soothing behaviour? (e.g., ripping of paper, rubbing of skin, etc.). YES / NO
   1. If YES, please specify: ______________________
6. In the PAST, has you child ever engaged in any self-soothing behaviour? (e.g., ripping of paper, rubbing of skin, etc.). YES / NO
   1. If YES, please specify: ______________________
7. Does your child CURRENTLY have recurrent, irresistible urges to pull out hair from their scalp, eyebrows or other areas of their body? YES / NO
   1. If YES, is your child diagnosed with trichotillomania (hair pulling disorder)
      YES / NO
8. In the PAST, has you child ever had recurrent, irresistible urges to pull out hair from their scalp, eyebrows or other areas of their body? YES / NO
9. Does your child ever engage in picking their skin (unaffected by skin condition) to the point that it causes damage? YES / NO
10. In the PAST, did your child frequently urinate in places other than the bathroom during the day? YES / NO
    1. If YES, did your child do this intentionally? YES / NO
11. Does your child CURRENTLY frequently urinate in places other than the bathroom during the day? YES / NO
    1. If YES, does your child do this intentionally? YES / NO
12. Does your child CURRENTLY have bowel movements in places other than the bathroom? YES / NO
    1. Do you feel that it is intentional? YES / NO
    2. If yes, do they play with it or smear it, etc. YES / NO
13. In the PAST, has your child had bowel movements in places other than the bathroom? YES / NO
    1. Do you feel that it was intentional? YES / NO
    2. If yes, did they play with it or smear it, etc. YES / NO

**Food and Eating Behaviours**

1. In the PAST, did your child take food without permission outside of meal times?

YES / NO

1. CURRENTLY, does your child take food without permission outside of meal times? YES / NO
2. Have you ever found your child hoarding food in a place other than the kitchen, such as their room or closet? YES / NO
3. During meals, does your child continue eating even after they are appear to be full? YES / NO
4. Has your child ever eaten to the point of feeling sick or throwing up? YES / NO
5. Do you feel you need to lock the fridge or pantry to keep your child from accessing food? YES / NO
6. During meals, does your child have an apparent lack of interest in eating or food?
   YES / NO
7. Is your child a picker eater?

YES / NO

- 1. If YES, do you think it is due to:
     1. Texture
     2. Colour
     3. Class of food (fruit vs vegetables)
     4. Other: ____________________
